# Ultra-processed foods and the nutritional transition among infants and young children: a radiography from Brazil

**DOI:** 10.1590/0102-311XEN118123

**Published:** 2023-10-23

**Authors:** Rafael Pérez-Escamilla

**Affiliations:** 1 Yale School of Public Health, New Haven, U.S.A.

By analyzing two national surveys - the *Brazilian National Survey on Demography and Health of Women and Children* (PNDS 2006) and the *Brazilian National Survey on Child Nutrition* (ENANI-2019) -, Castro et al. ^1^ found that while the nutrition transition advances in Brazil, there were encouraging improvements in exclusive breastfeeding and several undernutrition outcomes from 2006 to 2019, including anemia and vitamin A deficiency, among children aged under five years. Furthermore, stunting remained relatively low over time (7%) ^1^. On the other hand, obesity rates increased in the same period, which is not surprising. In 2019, the alarming prevalence of 88% of children aged under five years consumption ultra-processed foods and 26% did not consume fruits or vegetables the day before the survey ^1^.

The extraordinarily high consumption of ultra-processed foods among young children combined with aggressive marketing from the food industry, creates a concerning scenario for the future of Brazil. This is because early exposure to these quite unhealthy foods is likely to affect the establishment of food preferences for the rest of one’s life and ultra-processed foods have been consistently associated with obesity and noncommunicable diseases (NCDs) including heart disease, type 2 diabetes, and several types of cancer, as well as poor mental health outcomes, including depression ^2^ ^,^ ^3^ ^,^ ^4^.

Equiplot graphs documented declines over time in undernutrition inequities as a function of maternal race/skin color, schooling level, and region of residence. Nevertheless, they show that these inequities persist ^1^. Unfortunately, this study could not discriminate trends as a function of time where different political administrations have ruled the country. This is important to consider when interpreting the findings as it is quite likely that the child nutrition outcomes may have deteriorated once a political group focused on undermining social and food security policies and programs governed from 2016-2023. In 2023 the party that had previously developed and implemented the progressive social policies returned to power.

A case in point is how the Jair Messias Bolsonaro’s administration, chose to eliminate the Brazilian National Food and Nutrition Security Council (CONSEA) and discarded or undermined several effective social protection policies ^5^ which may partly explain why after severe household food insecurity decreased by 53% from 2003-2013 ^5^, it then steadily increased starting in 2013 and in the first months of the COVID-19 pandemic in 2020 ^5^. This was followed by an impressive increase during the pandemic ^6^ ^,^ ^7^. Indeed in 2020, the first year of the pandemic, 19 million Brazilians experienced severe food insecurity, almost twice the figure of 2018, 10 million people ^6^. Furthermore in 2021-2022 an alarming 33 million Brazilians experienced severe food insecurity ^7^. During all periods, households that were low-income, with children, headed by women, and with black people, were disproportionately affected by severe food insecurity ^5^ ^,^ ^6^ ^,^ ^7^.

A positive aspect in Brazil is that it counts with exemplar evidence-based dietary guidelines for infants and young children ^8^ that strongly recommend breastfeeding practices based on World Health Organization/United Nations Children’s Fund (WHO/UNICEF) recommendations, timely introduction of nutritious, healthy, and safe complementary foods, and avoidance of ultra-processed foods including sugar-sweetened beverages. The guidelines strongly endorse responsive feeding principles and the transition into healthy family meals by the time the child is two years old. They also emphasize home cooking, food safety, and the link between healthy foods that are produced following sustainable practices and environmental sustainability which is key for the health of our planet ^9^.

Furthermore, the Brazilian dietary guidelines specifically call for protecting families with children against predatory marketing practices by the food and beverages industry ^9^. UNICEF recently released recommendations on how to do so for commercial infant formulas and other ultra-processed foods as a key step to drastically reform food systems in the context of the rights of children ^10^. UNICEF recommends not allowing the food and beverages industry to engage in public policy making, and not partnering with companies that violate the International Code of Marketing Breast-milk Substitutes and related World Health Assembly resolutions ^10^. These recommendations are very responsive to the calls to action from the 2023 Lancet Series on Breastfeeding and the 2023 Lancet Series on Commercial Determinants of Health that exposed the sophisticated marketing playbooks from diverse powerful industries to entice consumers to consume unhealthy products, such as ultra-processed foods, throughout life course - including the crucial first 1,000 days of life.

Moving forward, Brazil needs to use a whole of society well-coordinated multi-sectoral approach that includes evidence-based advocacy, political will reflected in legislation, regulatory policies, and strong incentives for making healthy foods and beverages the default system, giving access to population to healthier foods since the beginning of life ^9^ ^,^ ^10^ ^,^ ^11^ ^,^ ^12^.

In conclusion, ENANI-2019 provides a very valuable but worrisome radiography of the malnutrition scenario in Brazil, especially regarding excessive consumption of ultra-processed foods and increases in childhood obesity. Brazil must enhance investments and efforts to protect, promote, and support healthy eating habits since early life including breastfeeding, complementary feeding, and transition into the family diets. Brazil should build upon its successes in the past at curving malnutrition via major social investments and equitable wealth redistribution ^5^ and it should continue refining its front of package warning labels ^13^ and sugar-sweetened beverages tax policies ^14^. Enhancing government subsidies for increasing access to fresh produce and other healthy foods combined with evidence-based nutrition education and counseling consistent with Brazil’s dietary guidelines should be a priority. Lastly, the sustainability of these efforts will depend on improved food and nutrition security governance through transparent and inclusive coordination across systems - including food, water, health, education, social protection, judicial systems - based on decentralized cost-effective program monitoring and evaluation systems that facilitate decision making from the municipal to the national level ^15^.
